# ARBs and risk of cancer: international and South African expert comment

**Published:** 2010-12

The suggestion from a recent meta–analysis that angiotensin receptor blockers (ARBs) are associated with an increase in new cancer occurrence but not cancer deaths,^1^ has resulted in the initiation of a safety review of this class of drugs by both the FDA (Federal Drug Administration) and the EMA (European Medicines Authority) in accordance with good regulatory practice. This was also advocated by Dr Steve Nissen in his editorial comment.^2^ In the interim, a review of the published meta–analysis plus input from Boehringer–Ingelheim is pertinent to clinical practice.

## Meta-analysis of randomised control trials^1^

This meta–analysis pooled the published randomised, controlled trials of ARBs and found that ARB use may be associated with a modest increased risk of new cancers – predominantly lung cancer. Patients who were randomly assigned to receive ARBs had an increased risk of new cancer occurrence compared with patients in the control groups (7.2 vs 6.0%, risk ratio 1.08, 95% CI, 1.01–1.15). When analysis was limited to those trials (LIFE, ONTARGET and TRANSCEND) where cancer was a pre–specified endpoint, the risk ratio was 1.11 (95% CI, 1.04–1.18, p = 0.001). The authors concluded that the findings of the meta–analysis warrant further investigation.

The meta–analysis reviewed 60 trials and included published and available FDA data from nine different trials (Table 1) to assess overall cancer risk and risk of specific solid–organ cancers associated with ARBs plus ACE inhibitor therapy, compared with ACE inhibitors alone. Cancer was a pre–specified endpoint of special interest in three of the five trials that included new cancer data for analysis of cancer occurrence (LIFE, ONTARGET and TRANSCEND).

**Table 1:** RANDOMISED CONTROLLED TRIALS OF ANGIOTENSIN RECEPTOR BLOCKERS THAT REPORTED CANCER DATA

	*Condition studied*	*Mean or median duration, years*	*Number of patients*	*Study drug*	*Control*	*History of cancer at baseline (%)*	
						*Study drug*	*Control*
Trials with data on new cancer, new specific solid organ cancers, and cancer death							
LIFE (2002)	Hypertension	4.8	9 193	Losartan up to 100 mg (n = 4605)	Atenolol up to 100 mg (n = 4588)	NA	NA
ONTARGET (2008)	Cardiovascular disease* or diabetes with end organ damage	4.7	25 620	Telmisartan 80 mg (n = 8542) or Telmisartan 80 mg + ramipril 5 mg (n = 8502)	Ramipril 5 mg (n = 8576)	6.3	6.3
TRANSCEND (2008)	ACE inhibitor intolerant patients with cardiovascular disease* or diabetes, with end organ damage	4.7	5 926	Telmisartan 80 mg (n = 2954)	Placebo (n = 2972)	4.9	4.9
PROFESS (2008)	Recent (< 90 days) ischaemic Stroke	2.5	20 332	Telmisartan 80 mg (n = 10146)	Placebo (n = 10186)	NA	NA
Trials with data on new specific solid organ cancers and cancer death							
CHARM Overall programme (2003)	Heart failure	3.1	7 599	Candesartan up to 32 mg (n = 3803)	Placebo (n = 3796)	7.1	6.4
Trials with new cancer data only							
TROPHY (2006)	Pre hypertension	3.6	787	Candesatran 16 mg (n = 391)	Placebo (n = 381)	NA	NA
Trials with cancer death data only							
VAL HEFT (2001)	Heart failure	1.9	5 010	Valsartan up to 120 mg twice daily (n = 2511)	Placebo (n = 2 499)	NA	NA
OPTIMAAL (2002)	Acute myocardial infarction	2.7	5 477	Losartan up to 50 mg daily (n = 2 744)	Captopril up to 50 mg three times daily	NA	NA
VALIANT (2003)	Acute myocardial infarction	2.1	14 626	Valsartan up to 80 mg twice daily (n = 4 885) or Valsartan up to 40 mg twice daily + captopril up to 25 mg three times daily (n = 4 862)	Captopril up to 25 mg three times daily (n = 4 879)	NA	NA

ACE = angiotensin converting enzyme. NA = not available. *Includes coronary, peripheral, or cerebrovascular disease

In the ONTARGET and TRANSCEND trials, information on the occurrence of malignancies was also collected prospectively in more detail than usual for trials of cardiovascular outcomes, thereby placing the spotlight on telmisartan which was the study drug in 30 014 (85.7%) of the ARB–treated patients included in the meta–analysis. The association of ARBs with the occurrence of solid–organ cancers, new lung, prostate and breast cancer from the meta–analysis is summarised in Table 2.

**Table 2:** SOLID–ORGAN CANCERS REPORTED IN RANDOMISED CONTROLLED TRIALS OF ANGIOTENSIN RECEPTOR BLOCKERS

*Lung cancer*	*ARB (%)*	*Control (%)*	*RR (95% CI)*	*%*	*p value*
All available trials
LIFE	29/4605 (0.6)	12/4588 (0.3)	2.41 (1.23–4.71)		0.01
CHARM Overall	31/3803 (0.8)	25/3796 (0.7)	1.24 (0.73–2.09)		0.43
TRANSCEND	35/2954 (1.2)	27/2972 (0.9)	1.30 (0.79–2.15)		0.30
ONTARGET	229/17044 (1.3)	101/8576 (1.2)	1.14 (0.90–1.44)		0.27
PROFESS	37/10016 (0.4)	30/10048 (0.3)	1.24 (0.77–2.00)		0.39
Meta analysis	361/38422 (0.9)	195/29980 (0.7)	1.25 (1.05–1.49)	6.6	0.01
With background ACE inhibitor treatment					
CHARM Added	12/1276 (0.9)	7/1272 (0.6)	1.71 (0.68–4.33)		0.26
ONTARGET (telmisartan + ramipril vs ramipril)	129/8502 (1.5)	101/8576 (1.2)	1.29 (0.99–1.67)		0.055
Meta analysis	141/9778 (1.4)	108/9848 (1.1)	1.32 (1.03–1.69)	0	0.031
Without background ACE inhibitor treatment					
LIFE	29/4605 (0.6)	12/4588 (0.3)	2.41 (1.23–4.71)		0.01
TRANSCEND	35/2954 (1.2)	27/2972 (0.9)	1.30 (0.79–2.15)		0.30
ONTARGET (telmisartan vs ramipril)	100/8542 (1.2)	101/8576 (1.2)	0.99 (0.76–1.31)		0.97
CHARM Alternative	10/1013 (1.0)	3/1015 (0.3)	3.34 (0.93–12.10)		0.066
Meta analysis	174/17114 (1.0)	143/17151 (0.8)	1.50 (0.93–2.41)	65	0.097
Prostate cancer*					
All available trials					
LIFE	58/2118 (2.7)	42/2112 (2.0)	1.38 (0.93–2.04)		0.11
CHARM Overall	32/2617 (1.2)	27/2582 (1.0)	1.17 (0.70–1.95)		0.55
TRANSCEND	35/1674 (1.2)	27/1705 (1.6)	1.32 (0.80–2.17)		0.27
ONTARGET	275/12544 (2.2)	128/6245 (2.0)	1.07 (0.87–1.32)		0.53
PROFESS	36/6455 (0.6)	32/6418 (0.5)	1.12 (0.70–1.80)		0.64
Meta analysis	436/25408 (1.7)	256/19062 (1.3)	1.15 (0.99–1.34)	0	0.076
With background ACE inhibitor treatment					
CHARM Added	7/1006 (0.7)	9.1000 (0.9)	0.77 (0.29–2.07)		0.61
ONTARGET (telmisartan + ramipril vs ramipril)	141/6252 (2.3)	128/6245 (2.0)	1.10 (0.87–1.39)		0.43
Meta–analysis	148/7258 (2.0)	137/7245 (1.9)	1.08 (0.86–1.36)	0	0.52
Without background ACE–inhibitor treatment					
LIFE	58/2118 (2.7)	42/2112 (2.0)	1.38 (0.93–2.04)		0.11
TRANSCEND	35/1674 (2.1)	27/1705 (1.6)	1.32 (0.80–2.17)		0.27
ONTARGET (telmisartan vs ramipril)	134/6292 (2.1)	128/6245 (2.0)	1.04 (0.82–1.32)		0.75
CHARM–Alternative	8/691 (1.2)	3/691 (0.4)	2.67 (0.71–10.01)		0.15
Meta–analysis	235/10775 (2.2)	200/10753 (1.9)	1.17 (0.97–1.41)		0.10
*Breast cancer†*					
All available trials					
LIFE	37/2487 (1.5)	36/2476 (1.5)	1.02 (0.65–1.61)		0.92
CHARM–Overall	17/1186 (1.4)	17/1214 (1.4)	1.02 (0.52–2.00)		0.95
TRANSCEND	20/1280 (1.6)	17/1267 (1.3)	1.16 (0.61–2.21)		0.64
ONTARGET	60/4500 (1.3)	34/2331 (1.5)	0.91 (0.60–1.39)		0.67
PROFESS	20/3561 (0.6)	15/3630 (0.4)	1.36 (0.70-2.65)		0.37
Meta–analysis	154/13014 (1.2)	119/10918 (1.1)	1.04 (0.82–1.32)	0	0.74
With background ACE inhibitor treatment					
ONTARGET (telmisartan + ramipril vs ramipril)	33/2250 (1.5)	34/2331 (1.5)	1.00 (0.61–1.66)		> 0.99
Without background ACE inhibitor treatment					
LIFE	37/2487 (1.5)	36/2476 (1.5)	1.02 (0.65–1.61)		0.93
TRANSCEND	20/1280 (1.6)	17/1267 (1.3)	1.16 (0.61–2.21)		0.64
ONTARGET (telmisartan vs ramipril)	27/2250 (1.2)	34/2331 (1.5)	0.83(0.50–1.36)		0.45
CHARM–Alternative	5/322 (1.6)	4/324 (1.2)	1.26 (0.34–4.64)		0.73
Meta–analysis	89/6339 (1.2)	91/6398 (1.4)	0.99 (0.74–1.32)	0	0.93

ARB = angiotensin receptors blocker. RR = risk ratio. ACE = angiotensin converting enzyme. *Analysis limited to men. †Analysis limited to women, all breast cancers were assumed to have occurred in women. Breast cancer data were not available for the CHARM–Added trial.

## Comment from Boehringer Ingelheim

Boehringer–Ingelheim commented that peer–reviewed meta–analyses of aggregate published data such as that of Sipahi et al.^1^ have their appropriate place in scientific research. However, these publications have well–recognised limitations, including the following:

- the analyses did not include the individual patient data for any of the trials
- the trials were not designed to explore cancer outcomes
- the adjudication of cancer diagnoses was not uniform among included studies
- the analyses did not consider the latency for the malignancies
- the analyses did not take into account the effect of gender, age, smoking or other known risk factors for malignancies.

Boehringer–Ingelheim had conducted a comprehensive internal safety data analysis including malignancy data, which has formed part of the submission package to regulatory bodies since 2008. This analysis includes patient level time–to–event data, which are presented as malignancies per 100 patient years, and no statistically significant difference was observed.

The *Cardiovascular Journal of Africa* obtained comment from Dr Carl Lombard, director of the Biostatistics Unit at the South African Medical Research Council (MRC) and Dr Adam Nosworthy, senior specialist physician and medical oncologist, University of the Witwatersrand, Charlotte Maxeke Johannesburg Academic Hospital, and clinical adviser to the South African Oncology Consortium. They reviewed the published and Boehringer–Ingelheim data for the Journal, and their comments follow.

## Comment from Dr Carl Lombard on Boehringer–Ingelheim analysis 

Systematic reviews, which evaluate different trials around the same question often lead to a formal pooled analysis of the relevant information through a meta-analysis. This is often a crude analysis since only the summarised data from trials are available from the publications.

The methodology of meta–analysis is well established and is a useful tool to pick up small signals of benefit or risk across trials with varying levels and direction of effect sizes. Sipahi et al.^1^ utilised the meta–analysis methodology to look at the risk of solid–organ cancers in randomised, controlled trials of angiotensin receptor blockers. The conclusions reached from this analysis are balanced and qualified and clearly outline the limitations and need for further analysis.

**Fig. 1:**
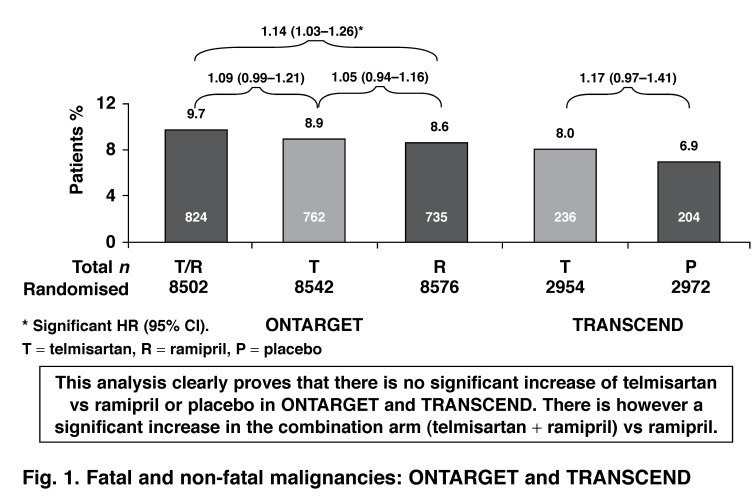
Fatal and non-fatal malignancies: ONTARGET and TRANSCEND

Three of the trials involved in this analysis used the Boehringer&ndash;Ingelheim ARB, telmisarton. Boehringer&ndash;Ingelheim has provided additional information involving patient&ndash;level information on the incidence and progression of cancers in the study participants of these trials (Fig. 1, Table 3).

**Table 3:** NUMBER OF PATIENTS WITH MALIGNANCIES BY ORGAN PER 100 PATIENT YEARS BY STUDY

	*ONTARGET*				*TRANSCEND*		
	*TR*	*T*	*R*	*ΔT–R*	*T*	*P*	*ΔT–P*
Randomised (n)	8502	8542	8576		2954	2972	
Patients with neoplasms^1^	2.14	1.96	1.88	0.08	1.72	1.48	0.25
Gastrointestinal	0.33	0.28	0.28	0	0.28	0.28	–0.01
Skin	0.39	0.34	0.37	–0.03	0.23	0.21	0.02
Prostate	0.36	0.33	0.31	0.01	0.25	0.2	0.05
Lung	0.32	0.23	0.25	–0.01	0.25	0.17	0.07
Genito–urinary	0.1	0.14	0.12	0.03	0.15	0.13	0.02
Blood	0.12	0.09	0.08	0.01	0.09	0.09	0
Breast	0.09	0.07	0.09	–0.02	0.14	0.13	0.01
Gynaecological	0.09	0.09	0.07	0.02	0.07	0.02	0.04
Head and neck	0.06	0.08	0.05	0.03	0.04	0.04	0
Metastases	0.05	0.05	0.03	0.02	0.02	0.01	0.01
Liver	0.05	0.06	0.05	0	0.04	0.03	0.01
Pancreas	0.05	0.05	0.05	0.01	0.06	0.05	0.01
CNS	0.03	0.03	0.04	–0.01	0.02	0.03	–0.01
Benign	0.01	0.01	0.01	0.01	0.01	0.01	0
NOS	0.03	0.03	0.02	0.01	0.03	0.01	0.01
Melanoma	0.04	0.04	0.04	0.01	0.01	0.03	–0.02
Endocrine	0.01	0.01	0.01	–0.01	0.01	0	0.01
Bone	0.01	0.01	0.01	0	0.01	0.01	0
Sarcoma	0.01	0.01	0	0	0.01	0.01	0.01
Abdominal	0.01	0.01	0	0	0	0.01	–0.01
Neuroendocrine	0	0	0	0	0	0	0

Δ represents the difference between treatment arms: + occurred more frequently and – less frequently in telmisartan group; T = telmisartan, R = ramipril, P = placebo.

The information provided across the three trials is differential and limited, which does not allow an appropriate pooled analysis across them. For the TRANSCEND and ONTARGET trials for example, the patient years of follow up is absent, whereas it is provided for the PRoFESS trial. The report reviews the results of the three trials separately.

Making approximate estimates for the patient years in TRANSCEND and ONTARGET from the information provided, and performing pooled analyses for overall malignancies, similar to that done by Sipahi et al.,^1^ the following was found:

1. In all three trials [PRoFESS (telmisartan vs placebo), TRANSCEND (telmisartan vs placebo), ONTARGET (telmisartan + ramipril, telmisartan vs ramipril)], the incidence rate ratio was 1.07; 95% CI: 0.99–1.14.
2. With background ACE inhibitor treatment [ONTARGET (telmisartan + ramipril vs ramipril)], the incidence rate ratio was 1.14; 95% CI: 1.03–1.16.
3. Without background ACE inhibitor treatment [PRoFESS (telmisartan vs placebo), TRANSCEND (telmisartan vs placebo), ONTARGET (telmisartan vs ramipril)], the incidence rate ratio was 1.03; 95% CI: 0.96–1.12.

These analyses still have some limitations in that they utilise only patient follow up and do not adjust for latency and other confounders. However, the comparisons are between large groups of patients that have been properly randomised, and with the same intensity of follow up and malignancy ascertainment.

From analysis 3 in which monotherapy telmisartan was compared to either placebo or ramipril, there is no evidence of risk for overall malignancy with regard to this product. With regard to the telmisartan/ ramipril combination arm, there is evidence of risk with regard to the incidence of overall malignancies.

The conclusion made by Boehringer– Ingelheim in their safety report is therefore objective: ‘There was a modest imbalance in malignancies seen in some of the recently completed cardiovascular outcome studies with telmisartan. This imbalance was primarily in the telmisartan/ramipril combination arm in ONTARGET, as opposed to monotherapy arms of telmisartan vs rampipril.’

However, the call for further analysis by Sipahi et al.^1^ still stands, since the safety report of Boehringer–Ingelheim does not utilise the full potential of the available individual–level data for pooled analyses.

## Comment from Dr Adam Nosworthy

The findings published by Sipahi et al.^1^ in the 14 June issue of the Lancet raise the concern of most doctors involved in clinical trials. (1) Do the treatments intended to offer benefit result in long–term harm to patients? (2) The latest trend of regulatory bodies to grant fast–track approval to new medications needs to be carefully reviewed.

In an attempt to offer patients the latest benefits, are we doing more harm than good in the long run? Having said that, the inferences made from the Sipahi et al.^1^ study that the risk of cancer is increased in patients taking ARBs is concerning to say the least.

The most important tenant of clinical trials is to determine which statistical endpoints need to be defined prior to commencing any study, and any *posthoc* analysis needs to be treated with the contempt that it deserves. To group a number of studies involving the ARBs (meta–analysis) and to extrapolate that there is an increased incidence of cancer in certain groups of patients is bad medicine and the outcomes of this report should not influence the use of these agents in patients.

The analysis is contradictory – there is an increase in lung cancer, which is claimed to be statistically significant in the group of patients receiving ARBs, yet the incidence of other cancers is decreased or the same. The group of patients that would typically be enrolled in these studies is firstly, a group of patients that are high risk for lung cancer, as they no doubt include a skewed bias in favour of smokers. All these factors would need to be included in the statistical design of the study prior to drawing these conclusions.

At this stage, I can find no reason to be concerned about the use of ARBs in patients. Far more reliable prospective, randomised data need to be presented prior to considering withdrawing this class of drug from the market.

It is rather ironic that there is a concern regarding a slight increase in cancer incidence in patients using ARBs in a retrospective analysis of numerous studies, yet a medication that is used widely and is known to have far greater impact on the development of breast cancer in women is prescribed in far greater numbers on a daily basis by doctors around the world – oestrogen replacement therapy – without as much as a mention in widely read medical journals!
